# 

**Published:** 1917-07-14

**Authors:** 


### HOSPITAL AND INSTITUTIONAL RESULTS.

**London Spectacle Mission Society.**—The annual income and expenditure of this Society are roughly £200. During last year £115 was spent on spectacles for needy persons. It is urged that expenses are necessarily higher as spectacles, like everything else, cost more in these costly times.

**Charing Cross Hospital.**—The report for general circulation is abbreviated, but a limited number of copies of the full report, including lists of subscribers, governors, etc., can be obtained on application. The feature of the income and expenditure account is the remarkable annual subscription total, £10,351. Although legacies amounted to £335 only, the income exceeded the expenditure by £10,455, and the Council was able to reduce the mortgage debt by £10,000.

**Gloucestershire Royal Infirmary.**—The number of in-patients has decreased by thirty-five, and out-patients show an increase of 172. There has been a falling-off in both donations and annual subscriptions. The financial position was much improved by Mr. William Long's legacy of £12,450, which has been invested in War Loan Stock. The ordinary income amounted to £6,935, and the ordinary expenditure to £9,900.

**Devonshire Hospital, Buxton.**—The cover of the report, designed by N. Sutcliffe, is artistic and attractive, showing nurses and wounded soldiers, with the domed hospital building as a background. The frontispiece consists of a charming reproduction of a crayon portrait of the Duchess of Portland, the chairman of the hospital. One thousand one hundred and thirty-five patients were admitted during the year, and the total of out-patients was larger than in any previous year. The total ordinary income was £18,746, and the total expenditure £16,858. Since the commencement of the war 2,433 soldiers suffering from rheumatism and allied diseases have been admitted.

**Dundee Royal Infirmary.**—The cost per occupied bed, £79 18s., is only £13 higher than in 1913; under the system of calculation adopted no deduction from the total ordinary expenditure has been made for the cost of out-patients. The total income (including £10,084 legacies and special donations) was £27,357, and the total expenditure £26,250.

Rates of Subscription: Twelve months, 6/6; Six months, 4/-; three months, 2/-, post free to any address in the United Kingdom. Abroad, Twelve months, 8/8; Six months, 5/-; Three months, 2/6, post free. **THE HOSPITAL "Index" to Volume LXI., October 1916 to March 1917, is now ready, price 2½d. per copy, post free.** Address The Manager, The Hospital, "The Hospital" Building, 28/29 Southampton Street, Strand, London, W.C. 2.

